# Data Mining *Mycobacterium tuberculosis* Pathogenic Gene Transcription Factors and Their Regulatory Network Nodes

**DOI:** 10.1155/2018/3079730

**Published:** 2018-03-14

**Authors:** Guangxin Yuan, Yu Bai, Yuhang Zhang, Guangyu Xu

**Affiliations:** ^1^College of Pharmacy, Beihua University, Jilin, Jilin 132013, China; ^2^Pharmaceutical College, Jilin Medical University, Jilin, Jilin 132011, China

## Abstract

Tuberculosis (TB) is one of the deadliest infectious diseases worldwide. In *Mycobacterium tuberculosis*, changes in gene expression are highly variable and involve many genes, so traditional single-gene screening of *M. tuberculosis* targets has been unable to meet the needs of clinical diagnosis. In this study, using the National Center for Biotechnology Information (NCBI) GEO Datasets, whole blood gene expression profile data were obtained in patients with active pulmonary tuberculosis. Linear model-experience Bayesian statistics using the Limma package in R combined with *t*-tests were applied for nonspecific filtration of the expression profile data, and the differentially expressed human genes were determined. Using DAVID and KEGG, the functional analysis of differentially expressed genes (GO analysis) and the analysis of signaling pathways were performed. Based on the differentially expressed gene, the transcriptional regulatory element databases (TRED) were integrated to construct the *M. tuberculosis* pathogenic gene regulatory network, and the correlation of the network genes with disease was analyzed with the DAVID online annotation tool. It was predicted that IL-6, JUN, and TP53, along with transcription factors SRC, TNF, and MAPK14, could regulate the immune response, with their function being extracellular region activity and protein binding during infection with *M. tuberculosis*.

## 1. Introduction

Tuberculosis (TB) is one of the deadliest infectious diseases in the world [1, 2]. One-third of the global population is estimated to be infected with *Mycobacterium tuberculosis*, and 5–10% of these patients have active disease [3]. Since the World Health Organization (WHO) announced TB as a global emergency, incidence and mortality of tuberculosis remain high, despite the large cost invested during this period [4]. A dominant reason behind this is the long-term latency of *M. tuberculosis* and lack of development of new, effective antituberculosis drugs. *M. tuberculosis* is an intracellular pathogen, and it is difficult for human antibodies to enter the cell from serum, and the cell-mediated immune response plays a very important role in either development of infection to active disease or containment of the pathogen. Therefore, the study of *M. tuberculosis* genes related to immunity and their regulatory network has become a focus of the current research.

Traditional single-gene screening of *M. tuberculosis* targets has been insufficient towards the development of new therapies [5]. Tuberculosis infectious disease is a complex disease involving the body's immune system, and traditional single-gene screening patterns do not fully and accurately reflect the occurrence, development, and pathogenesis of infectious diseases [6]. Therefore, integrating multidisciplinary approaches to construct an active pulmonary TB immune response gene regulatory network and then utilizing high-throughput screening to look for diagnostic targets in body fluids would add significant understanding of the regulatory network involved in the immune response to *M. tuberculosis* [7]. This strategy may lay a foundation for the early clinical diagnosis of TB and the development of new effective drugs for TB treatment [8].

In this study, using the National Center for Biotechnology Information (NCBI) GEO Datasets [9], the fluid (whole blood) gene expression profile data were obtained in patients with active pulmonary tuberculosis. Linear model-experience Bayesian statistics using the Limma package in R combined with *t*-tests were applied for nonspecific filtration of the expression profile data [10], and the differentially expressed genes were screened out. Using DAVID (Database for Annotation Visualization and Integrated Discovery) [11] and KEGG (Kyoto Encyclopedia of Genes and Genomes) [12], the functional analysis of differentially expressed genes (GO analysis) [13] and the analysis of signaling pathways (pathway analysis) [14] were performed. Based on the differentially expressed gene set, the transcriptional regulatory element databases (TRED) [15] were integrated to construct the *M. tuberculosis* pathogenic gene regulatory network, and the correlation of the network genes with disease was analyzed with the DAVID online annotation tool.

## 2. Method

### 2.1. Gene Chip Array Data

The gene chip array data were retrieved from GEO Datasets of the National Center for Biotechnology Information (NCBI, USA) database using keywords “tuberculosis” and “*Homo sapiens*” [porgn:_txid9606] [9]. Data from Affymetrix gene chips from human subjects infected with *M. tuberculosis* and with the original CEL documents available were selected.

We chose gene expression profile of GSE16250 from GEO database, which was a public and freely available database. GSE20050 was based on agilent GPL1352 platform. The GSE52819 dataset included 12 samples, containing 6 TB samples and 6 healthy samples. We also downloaded the Series Matrix File of GSE54992 from GEO database (Table 1).

### 2.2. Chip Data Processing

The Affymetrix Expression Console software tool was applied for the background correction of the collected chip data and the conversion of probe fluorescence values into gene expression values [16]. The Affymetrix Transcriptome Analysis Console was used for the logarithmic calculation and standardization of the chip data. The difference in mRNA expression between the chips in healthy people and those infected with *M. tuberculosis* was compared by SAM, and the genes with fold change > 2.0 or <−2.0 and *p* value < 0.05 were selected as differentially expressed genes. The differentially expressed genes were further screened by a Venn diagram; considering the differences between the chip platforms, the genes that overlapped with three or more than three platforms were selected.

### 2.3. Transcription Factor and Target Gene Screening

Transcription factor and target gene screening analysis was carried out at the TRED website http://rulai.cshl.edu/TRED [15].

### 2.4. Regulatory Network Diagram

Eighteen transcription factors (TF) and 887 target genes were predicted to a combined total of 887 TF-to-target pairs. The relation obtained from the analysis on the differential coexpression was mapped to the human transcription factors and target gene pairs to obtain transcription regulation pairs. Finally, Cytoscape software was used for plotting [17].

### 2.5. Gene Function Annotation Analysis

This analysis was carried out using DAVID database at website. 1075 genes were included in the analysis, and the human genome was used as the source of background genes [11].

### 2.6. Analysis of Differentially Expressed Gene Functions

Based on the NCBI Gene Ontology database, the GO annotation of these genes was performed to obtain all GO terms which genes were involved [13]. Fisher's exact test and *χ*^2^ test were used to calculate the significance level and misdiagnosis rate of each GO, and the *p* value was calibrated with the misdiagnosis rate, thereby screening out the GO significance reflected by the differentially expressed genes (*p* < 0.05). Using the European Bioinformatics Institute (EBI) database, the experimental results were manually analyzed.

### 2.7. Analysis of KEGG Gene Pathways

All pathways used in this study were downloaded from KEGG databases. In total, 78 relevant KEGG pathways were downloaded from http://www.genome.jp/kegg/ in September 2016 [12].

## 3. Results

### 3.1. Gene Chip Data

All the chip data in this study were taken from GEO Datasets of the National Center for Biotechnology Information (NCBI) database. The gene expression data of humans infected with *M. tuberculosis* based on Affymetrix gene chips with the original CEL document were selected, and 4 groups of gene chips were determined, as shown in Table 1.

These 4 groups of chips included those whose chip number of chips was 6, 7, 12, and 21, and each chip group was divided into two subgroups, a healthy subgroup and TB patient subgroup.

### 3.2. Chip Data Processing

**Figure 1:**
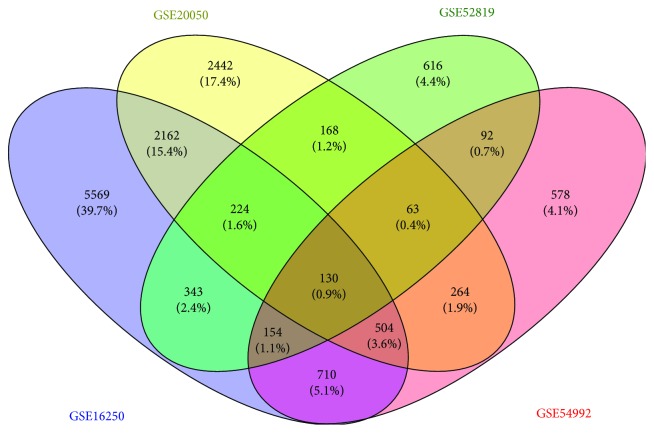
Screening results of genes overlapping ≥3 platforms. The blue part is GSE16250, the yellow part is GSE20050, the green part is GSE52819, and the pink part is GSE54992.

The logarithm and standardization of chip data were conducted using Affymetrix's Transcriptome Analysis Console, and the differentially expressed mRNA between the chips of healthy people (healthy group) and those infected with *M. tuberculosis* (TB patient group) was compared by the SAM method to determine differentially expressed genes. The number of differentially expressed genes of each platforms was 5569, 2442, 616, and 578, respectively. These genes were cross-screened again, and the results showed that there were 130 differentially expressed genes on the 4 platforms, 224, 63, 504, and 154 on three platforms, respectively, with a total of 1075 genes (Figure 1).

### 3.3. TB Transcription Factor Regulatory Network Diagram

Using TRED (Transcriptional Regulatory Element Database) database (http://rulai.cshl.edu/TRED), transcription factors interacting among the 1075 expressed genes were predicted and analyzed, and 887 genes were found to correspond to 18 transcription factors (Table 2).

**Figure 2:**
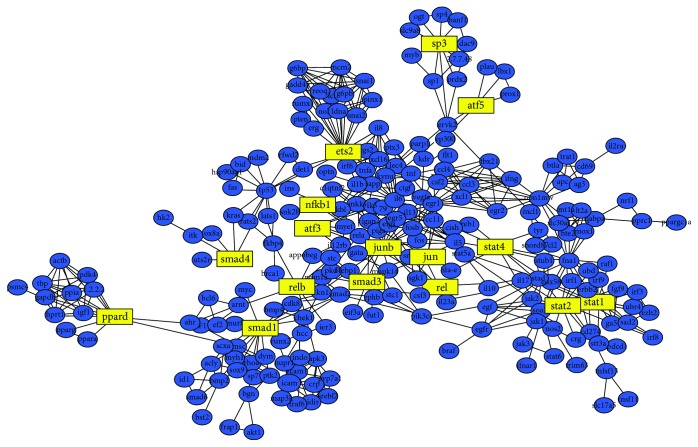
Regulatory network diagram of the 18 transcription factors found in TB patients. The yellow parts represent 18 transcription factors, and the blue parts are their corresponding target genes.

The regulatory network diagram of *M. tuberculosis* pathogenic gene transcription factors of the 18 transcription factors and their corresponding target genes was mapped with Cytoscape software (Figure 2).

Sixteen target genes were regulated by more than 1 transcription factor (Table 3), of which the target genes regulated by the most transcription factors were IL-6, MAPK14, RELA, and FOS, regulated by 4, 3, 3, and 3 TFs, respectively.

### 3.4. Network Nodes

As shown in Figure 2, statistical analysis of network nodes in the transcription factor regulatory network diagram found more than 10 nodes that had 44 genes, of which there were 20 nodes that had more than 4 genes, including IL-6, ETS2, TNF, and OPN1MW (Table 4). Furthermore, IL-6 was regulated by the most transcription factors, and genes such as JUN and IL-5 were also closely related to the regulation of *M. tuberculosis* pathogenic gene transcription factors.

### 3.5. GO Function Annotation Analysis

GO function annotation for 1075 different genes was carried out and sorted based on the *p* values, and 10 pathways were determined (Table 5). These 10 pathways were closely related to the disease, and the TB pathway was ranked in the top five of the pathways found.

## 4. Discussion

Tuberculosis caused by the infection of *M. tuberculosis* remains a threat to human health throughout the world. Currently, about 2 billion (1/3) people worldwide are infected with *M. tuberculosis*, but only 10% will develop active tuberculosis and the remaining 90% will make the disease to curb due to the body's own well-developed immune system [18]. Our previous study found that the immune response mechanism of host cells plays a crucial role in the process of microbial infection [19]. The disease process of active tuberculosis is transformed into the active stage and depends not only on the adaptive changes of mycelium caused by genes regulation but also on the transcription factor regulation network of the host's immune system [20]. To understand the defense mechanism of the host immune system against *M. tuberculosis* infection will provide a theoretical basis for the progress and prevention of active tuberculosis.

In this study, utilizing GEO Datasets from the NCBI database and using the Affymetrix Transcriptome Analysis Console, the logarithm transformation, and standardization of chip data were carried out, and 1075 differentially expressed *M. tuberculosis* pathogenic genes were found. The TRED database was used to predict and analyze these 1075 genes, and as a result, 18 transcription factors and 887 genes were found, and an *M. tuberculosis* pathogenic gene transcription regulation network diagram was constructed (Figure 2). Through a series of statistical analysis based on Figure 2, we found that many of these key factors are closely related to the human immune system.

As shown in Figure 2, there were 2 transcription factors that corresponded highly to the target genes, namely, JUN (236 target genes) and NFKB1 (221 target genes), whereas the other transcription factors corresponded to less than 50 target genes. JUN is closely linked with SLE (systemic lupus erythematosus), and SLE is a typical autoimmune disease involving multiple organs and systems. Doniz-Padilla et al. [21] found that JUN expression level in peripheral blood mononuclear cells (PBMCs) of patients with SLE was significantly higher than in individuals in the control group. JUN might play an important role in the transcriptional regulation of FCGR2B promoter activity, while FCGR2B has been shown to be closely associated with the pathogenesis of SLE [22]. These results suggest that c-JUN may be involved in the pathogenesis of SLE. NFKB1 gene is one of members in NF-*κ*B family of widespread transcription factors. It plays an important role in the immune response, inflammation, and cell growth and development.

The statistics of genes regulated by the transcription factors in Figure 2 also showed that the target genes regulated by more than 3 transcription factors were IL-6, MAPK14, RELA, and FOS; JUN was also regulated by the 2 transcription factors. Studies have shown that 4 genes, including IL-6, MAPK14, RELA, and FOS, are closely related to the immune system. IL-6 is a B cell stimulating factor and can also activate macrophages. It is produced not only by T cells but also by macrophages, fibroblasts, epithelial cells, and other cells outside the lymphatic system. IL-6 can provide a non-T cell-dependent response to acute and chronic infection with *M. tuberculosis*. IL-6 can act on multiple cells, such as B cells, liver cells, hybridoma cells, and plasma cells, and enhance immune resistance through its proinflammatory activity and the effect on the production of the other cytokines. Different from other type-2 cytokines, IL-6 not only is essential for the activation of T cells to secret IFN-*γ* but also presents as the main molecule to induce and protect T cells during *M. tuberculosis* infection and strengthen activities of IFN-*γ* [23]. Ladel et al. [24] found that after infection with *M. tuberculosis*, the production of IFN-*γ* in IL-6-deficient mice was reduced and their lifetime was shortened compared with wild-type mice. Although many studies have shown that IL-6 could induce a protective immune response against *M. tuberculosis* infection, some reports have demonstrated that IL-6 also has an adverse effect to inhibit the responsiveness of macrophages to IFN-*γ* [25]. MAPK14 gene is a member of mitogen-activated protein kinase (MAPK) family, and the MAPK cascade is a ubiquitous intracellular serine/threonine protein kinase super family. Thus, an important substance that can transmit the cytoplasmic signaling into the nucleus and cause the changes in the nucleus closely linked with the cell apoptosis and proliferation, tumor genesis, and oxidative stress injury of intestinal epithelial cells [26].

*M. tuberculosis* can exist in a nonproliferative state in the body of its host, thus resulting in a delayed course of the disease and a persistent recurrence of infection. The persistence of *M. tuberculosis* in macrophages may exert a huge survival pressure from bactericidal components in the cells. During its long evolutionary process and coevolution with humans, *M. tuberculosis* has developed a unique signal transmission system to ensure that it can start different response elements in different infection cycles to regulate the expression of genes for the adaptation to the environment. Among the numerous response (stringent response) regulatory elements, RELA plays a key role [27–29]. Studies have showed that the resistance and tolerance of bacteria to environmental pressure are dependent on RELA and the resulting ppGpp (P) signal can mediate bacterial resistance to antibiotics, UV-resistant survival, and DNA damage repair. *M. tuberculosis* with inactivated RELA cannot survive for a long time in an environment with the abovementioned types of pressure [30]. There are some differences in the morphology and chromaticity between long-term persisters and *M. tuberculosis* active propagules; persisters isolated from the lungs show extremely close manifestations to those under conditions of pressure, especially lack of nutrition, which is completely consistent with the starting condition of RELA. Another study showed that RELA is closely related to the bacterial morphology and colony formation caused by changes in the cell wall of mycobacterium, which is important for the formation of *M. tuberculosis* L-forms, namely, cell wall-deficient *M. tuberculosis*. RELA is thus a good target against persistent tuberculosis infection [31, 32].

FOS gene is a member of FOS family, along with JUN family members and other activated transcription factor protein family members including activator protein 1 (AP-1). AP-1 is an important transcription regulation factor in the nucleus, playing an important role in the transduction process of many signals in the body, and is the intersection of a series of nuclear cellular signal transduction pathways [8]. FOS can transmit extracellular stimulatory signals through the upstream signaling pathway to AP-1 and then activate the AP-1 to regulate the expression of a series of downstream target genes with AP-1 DNA recognition sites, that is, binding the specific AP-1 DNA recognition site 5′-TGA (G/C TCA-3′), also known as the TPA response element (TRE), to allow the body to make an adaptive response to external stimuli. In addition, AP-1 plays an important regulation role in SLE.

**Figure 3:**
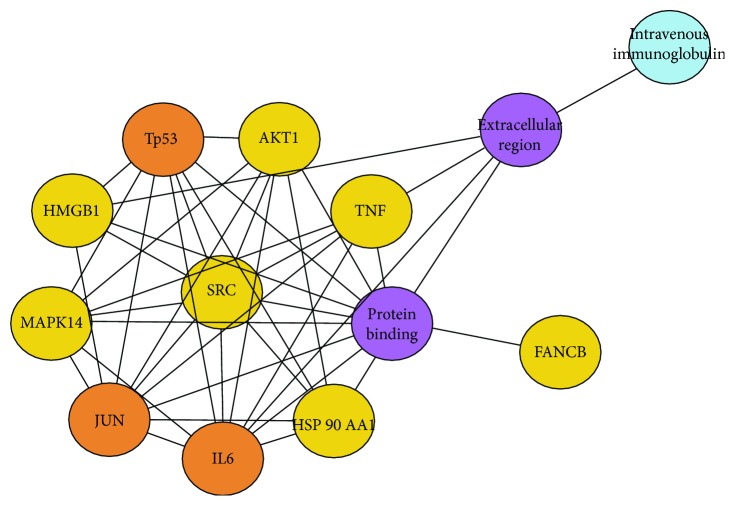
Network diagram of the relationship between network nodes. The blue part is the drug, the two purple parts are Gene Ontology, the yellow parts are the transcription factors, and the brown parts are the network nodes.

The statistics of network nodes is shown in Figure 2. The results showed that there were 4 genes with more than 20 network nodes, respectively, IL-6, ETS2, TNF, and OPN1MW (Table 4). As discussed previously, IL-6 is a gene regulated by transcription factors and JUN and IL-5 are also the genes regulated by transcription factors and the network node. Based on analysis of previous publications and analysis of all the network nodes, we suggest a certain portion between most of the networks (Figure 3), also closely related to the transcription factor SRC. Many of these nodes were targets of intravenous immunoglobulin drugs (Figure 3), and it is well known that intravenous immunoglobulin can rapidly raise the level of the IgG in the blood of recipients to enhance the body's ability to fight infections and regulate immune function. We also found that the major GO terms of these network nodes were in the extracellular region and were protein binding. Therefore, we presume that the network nodes IL-6, JUN, and TP53, along with transcription factors SRC, TNF, and MAPK14, can regulate the body's immune function, and their gene function should be mainly acting in the extracellular region and be protein binding.

## 5. Conclusions

In summary, 1075 genes were screened out through GEO database and a transcription factor regulation network diagram was constructed. Through the statistical analysis of the transcription factors and network nodes of their regulatory network diagram, we speculate that IL-6, JUN, and TP53, along with transcription factors SRC, TNF, and MAPK14, could regulate the human immune function, evading the attack on the host immune system and ultimately leading to the existence of *M. tuberculosis* in a nonproliferative state in the body of the host, thereby resulting in a delayed course of the disease and a recurrent persistent infection.

## Figures and Tables

**Table 1:** TB chip data.

Dataset ID	Sample ID	Sample number	Control sample number	Disease sample number	Platforms	Organism	Submission date	Manufacturer
GSE16250 [33]	GSM409134–GSM409139	6	3	3	GPL570	*Homo sapiens*	May 27, 2009	Affymetrix
GSE20050 [34]	GSM501249–GSM501255	7	2	5	GPL1352	*Homo sapiens*	July 19, 2004	Affymetrix
GSE52819 [35]	GSM1276660–GSM1276662 GSM1276666–GSM1276668	12	6	6	GPL6244	*Homo sapiens*	Dec. 05, 2007	Affymetrix
GSE54992 [36]	GSM1327526–GSM1327531 GSM1327533, GSM1327535, GSM1327540–GSM1327542, GSM1327544, GSM1327546, GSM1327549, GSM1327550	21	11	10	GPL570	*Homo sapiens*	Nov. 07, 2003	Affymetrix

**Table 2:** 18 transcription factors and their target genes.

TF	Target gene number	Gene ID	Description
SMAD1	30	4086	SMAD family member 1
SMAD4	39	4089	SMAD family member 4
MYBL1	7	4603	MYB proto-oncogene like 1
STAT1	57	6772	Signal transducer and activator of transcription 1
JUNb	10	3726	JUNb proto-oncogene, AP-1 transcription factor subunit
ETS2ETS2	64	2114	ETS proto-oncogene 2, transcription factor
NF-*κ*B1	211	4790	Nuclear factor kappa B subunit 1
NF-*κ*B2	8	4791	Nuclear factor kappa B subunit
ATF3	12	467	Activating transcription factor 3
ATF5	1	22809	Activating transcription factor 5
SP3	95	6670	Sp3 transcription factor
JUN	236	3725	JUN proto-oncogene, AP-1 transcription factor subunit
PPARD	27	5467	Peroxisome proliferator activated receptor delta
STAT2	2	6773	Signal transducer and activator of transcription 2
REL	26	5966	REL proto-oncogene, NF-*κ*B subunit
RELB	7	5971	RELB proto-oncogene, NF-*κ*B subunit
STAT4	8	6775	Signal transducer and activator of transcription 4
SMAD3	47	4088	SMAD family member 3

**Table 3:** Genes regulated by transcription factors.

Genes regulated by transcription factors	Number of transcription factors regulating the target genes	Network node (Figure 2)
IL-6	4	30
MAPK14	3	—
RELA	3	11
FOS	3	13
JUN	2	13
NF-kappa 1	2	14
IL-1b	2	16
COL11	2	—
EGR1	2	18
EGR3	2	—
FOSb	2	—
IL-13	2	—
IL-5	2	10
SGK1	2	—
SMAD2	2	10
SRC	2	—

**Table 4:** Network node statistics.

Gene	Network node
IL-6	30
ETS2	29
TNF	25
OPN1MW	21
IFNAL	19
STAT1	18
SMAD1	18
EGR1	18
JUNb	17
IL-1b	16
STAT3	14
NF-kappa B	14
STAT2	13
JUN	13
FOS	13
TP53	12
TNF*α*	12
PTX3	12
PTGS2	12
PPARD	12
KYNU	12
IRF6	12
IL-8	12
CXCL16	12
CLEC4E	12
ATF3	12
STAT4	11
SNAI2	11
SNAIL	11
SMAD3	11
SLC2A1	11
RELA	11
REC8	11
PINX1	11
NOL3	11
MCM2	11
lLDHA	11
IGFBP3	11
GADD45G	11
G6PD	11
SP3	10
SMAD2	10
RELB	10
IL-5	10

**Table 5:** GO function annotation of differentially expressed genes.

Pathway	Gene number	*p* value	Benjamini
Influenza A	40	2.6*E* − 10	7.2*E* − 8
Osteoclast differentiation	30	4.8*E* − 9	6.6*E* − 7
Legionellosis	19	3.1*E* − 8	2.8*E* − 6
NF-kappa B signaling pathway	24	5.4*E* − 8	3.7*E* − 6
Tuberculosis	36	6.9*E* − 8	3.8*E* − 6
TNF signaling pathway	26	1.6*E* − 7	7.5*E* − 6
Hepatitis B	30	7.4*E* − 7	2.9*E* − 5
Herpes simplex infection	34	1.5*E* − 6	5.2*E* − 5
Cytokine-cytokine receptor interaction	39	2.3*E* − 6	7.0*E* − 5
RIG-I-like receptor signaling pathway	19	2.4*E* − 6	6.5*E* − 5

## References

[1] Dutta N. K., Mehra S., Didier P. J. (2010). Genetic requirements for the survival of tubercle bacilli in primates. *The Journal of Infectious Diseases*.

[2] Saramba M. I., Zhao D. (2016). A perspective of the diagnosis and management of congenital tuberculosis. *Journal of Pathogens*.

[3] Getahun H., Matteelli A., Abubakar I. (2015). Management of latent *Mycobacterium tuberculosis* infection: WHO guidelines for low tuberculosis burden countries. *European Respiratory Journal*.

[4] Weiner J., Kaufmann S. H. (2016). High-throughput and computational approaches for diagnostic and prognostic host tuberculosis biomarkers. *International Journal of Infectious Diseases*.

[5] Hong-Geller E., Micheva-Viteva S. N. (2010). Functional gene discovery using RNA interference-based genomic screens to combat pathogen infection. *Current Drug Discovery Technologies*.

[6] Vasava M. S., Bhoi M. N., Rathwa S. K., Borad M. A., Nair S. G., Patel H. D. (2017). Drug development against tuberculosis: past, present and future. *The Indian Journal of Tuberculosis*.

[7] Zhou T. T. (2011). Network systems biology for targeted cancer therapies. *Chinese Journal of Cancer*.

[8] Hmama Z., Pena-Diaz S., Joseph S., Av-Gay Y. (2015). Immunoevasion and immunosuppression of the macrophage by *Mycobacterium tuberculosis*. *Immunological Reviews*.

[9] Davis S., Meltzer P. S. (2007). GEOquery: a bridge between the Gene Expression Omnibus (GEO) and BioConductor. *Bioinformatics*.

[10] Morrissey E. R., Juarez M. A., Denby K. J., Burroughs N. J. (2011). Inferring the time-invariant topology of a nonlinear sparse gene regulatory network using fully Bayesian spline autoregression. *Biostatistics*.

[11] Huang Da W., Sherman B. T., Lempicki R. A. (2009). Systematic and integrative analysis of large gene lists using DAVID bioinformatics resources. *Nature Protocols*.

[12] Ogata H., Goto S., Sato K., Fujibuchi W., Bono H., Kanehisa M. (1999). KEGG: Kyoto Encyclopedia of Genes and Genomes. *Nucleic Acids Research*.

[13] Ashburner M., Ball C. A., Blake J. A. (2000). Gene ontology: tool for the unification of biology. The Gene Ontology Consortium. *Nature Genetics*.

[14] Xu G., Ni Z., Shi Y. (2014). Screening essential genes of *Mycobacterium tuberculosis* with the pathway enrichment method. *Molecular Biology Reports*.

[15] Zhao F., Xuan Z., Liu L., Zhang M. Q. (2005). TRED: a Transcriptional Regulatory Element Database and a platform for *in silico* gene regulation studies. *Nucleic Acids Research*.

[16] Lu J., Lee J. C., Salit M. L., Cam M. C. (2007). Transcript-based redefinition of grouped oligonucleotide probe sets using AceView: high-resolution annotation for microarrays. *BMC Bioinformatics*.

[17] Shannon P., Markiel A., Ozier O. (2003). Cytoscape: a software environment for integrated models of biomolecular interaction networks. *Genome Research*.

[18] Mayer-Barber K. D., Andrade B. B., Oland S. D. (2014). Host-directed therapy of tuberculosis based on interleukin-1 and type I interferon crosstalk. *Nature*.

[19] Banuls A. L., Sanou A., Anh N. T., Godreuil S. (2015). *Mycobacterium tuberculosis*: ecology and evolution of a human bacterium. *Journal of Medical Microbiology*.

[20] Blischak J. D., Tailleux L., Mitrano A., Barreiro L. B., Gilad Y. (2015). Mycobacterial infection induces a specific human innate immune response. *Scientific Reports*.

[21] Doniz-Padilla L., Martinez-Jimenez V., Nino-Moreno P. (2011). Expression and function of Cbl-b in T cells from patients with systemic lupus erythematosus, and detection of the 2126 A/G Cblb gene polymorphism in the Mexican mestizo population. *Lupus*.

[22] Olferiev M., Masuda E., Tanaka S., Blank M. C., Pricop L. (2007). The role of activating protein 1 in the transcriptional regulation of the human FCGR2B promoter mediated by the -343 G → C polymorphism associated with systemic lupus erythematosus. *Journal of Biological Chemistry*.

[23] Leal I. S., Smedegard B., Andersen P., Appelberg R. (1999). Interleukin-6 and interleukin-12 participate in induction of a type 1 protective T-cell response during vaccination with a tuberculosis subunit vaccine. *Infection and Immunity*.

[24] Ladel C. H., Blum C., Dreher A., Reifenberg K., Kopf M., Kaufmann S. H. (1997). Lethal tuberculosis in interleukin-6-deficient mutant mice. *Infection and Immunity*.

[25] Saunders B. M., Frank A. A., Orme I. M., Cooper A. M. (2000). Interleukin-6 induces early gamma interferon production in the infected lung but is not required for generation of specific immunity to *Mycobacterium tuberculosis* infection. *Infection and Immunity*.

[26] Zhou Y., Wang Q., Mark Evers B., Chung D. H. (2006). Oxidative stress-induced intestinal epithelial cell apoptosis is mediated by p38 MAPK. *Biochemical and Biophysical Research Communications*.

[27] Sajish M., Kalayil S., Verma S. K., Nandicoori V. K., Prakash B. (2009). The significance of EXDD and RXKD motif conservation in Rel proteins. *Journal of Biological Chemistry*.

[28] Rifat D., Bishai W. R., Karakousis P. C. (2009). Phosphate depletion: a novel trigger for *Mycobacterium tuberculosis* persistence. *The Journal of Infectious Diseases*.

[29] Lu L. D., Sun Q., Fan X. Y., Zhong Y., Yao Y. F., Zhao G. P. (2010). Mycobacterial MazG is a novel NTP pyrophosphohydrolase involved in oxidative stress response. *Journal of Biological Chemistry*.

[30] Dhiman R., Raje M., Majumdar S. (2007). Differential expression of NF-*κ*B in mycobacteria infected THP-1 affects apoptosis. *Biochimica et Biophysica Acta*.

[31] Murphy D. J., Brown J. R. (2007). Identification of gene targets against dormant phase *Mycobacterium tuberculosis* infections. *BMC Infect ious Diseases*.

[32] Park K. T., Dahl J. L., Bannantine J. P. (2008). Demonstration of allelic exchange in the slow-growing bacterium *Mycobacterium avium* subsp. paratuberculosis, and generation of mutants with deletions at the *pknG*, *relA*, and *lsr*2 loci. *Applied and Environmental Microbiology*.

[33] Reyes N., Bettin A., Reyes I., Geliebter J. (2015). Microarray analysis of the in vitro granulomatous response to *Mycobacterium tuberculosis* H37Ra. *Colombia Médica*.

[34] Kim M. J., Wainwright H. C., Locketz M. (2010). Caseation of human tuberculosis granulomas correlates with elevated host lipid metabolism. *EMBO Molecular Medicine*.

[35] Verway M., Bouttier M., Wang T. T. (2013). Vitamin D induces interleukin-1*β* expression: paracrine macrophage epithelial signaling controls *M. tuberculosis* infection. *PLoS Pathogens*.

[36] Cai Y., Yang Q., Tang Y. (2014). Increased complement C1q level marks active disease in human tuberculosis. *PLoS One*.

